# The Effect of Osmotherapy and Tight Control of Acidosis on Early Graft Function among Deceased-Donor Kidney Transplant Recipients: A Randomized Controlled Trial

**Published:** 2017-02-01

**Authors:** F. Etezadi, A. H. Najafi Abrandabadi, J. Motaharinia, M. Mojtahedzadeh, P. Pourfakhr, M. R. Khajavi, S. Gooran, R. Shariat Moharari, S. Dehghani

**Affiliations:** 1 *Department of Anesthesiology and Critical Care, Sina Hospital, Tehran University of Medical Sciences, Tehran, Iran *; 2 *Department of Pharmacotherapy, Faculty of Pharmacy, Tehran University of Medical Sciences, Tehran, Iran*; 3 *Urology Research Center, Tehran University of Medical Sciences, Tehran, Iran*

**Keywords:** Kidney transplantation, Deceased-donor, Acidosis, Bicarbonates, Saline solution, hypertonic

## Abstract

**Background:**

Reperfusion injury and the acid-base status of the transplant are important factors affecting post-transplantation graft function.

**Objective:**

We hypothesized that infusing hypertonic saline (HS) or tight control of acid-base status of the blood rushing through renal graft using sodium bicarbonate may have beneficial effects on early graft function.

**Methods:**

Candidates for deceased-donor kidney transplant were randomized into three groups. HS group (n=33) received 50 mL/kg normal saline (NS) titrated during operation plus 4 mL/kg of 5% HS just within graft reperfusion phase; bicarbonate group (n=37) was administered 60 mL/kg NS while their metabolic acidosis (base excess ≤5 mEq/L) was tightly corrected every 30 min with sodium bicarbonate; and a control group (n=36) that received 60 mL/kg normal saline while they were administered sodium bicarbonate only, if they encountered severe metabolic acidosis (base excess ≤15 mEq/L). The primary outcome was defined as early post-operative renal function evaluated based on serial serum creatinine levels. The study was registered in Iranian Registry of Clinical Trials (IRCT2013122815841N19).

**Results:**

Post-operative early graft function improved significantly during the first 3 days in the intervention groups (p<0.05). However, that beneficial effect no longer remained at the same level after the day four.

**Conclusion:**

Timely administration of HS or tight control of metabolic acidosis with sodium bicarbonate infusion improve early renal function during renal transplant surgery.

## INTRODUCTION

Warm and cold ischemias are the two inevitable phenomena that affect deceased-donor kidney transplant. Although reperfusion is essential to stop ischemic injury, it may lead to further damage to the graft, a process the so-called “ischemia-reperfusion injury” (IRI) [1]. Infiltration of neutrophils, macrophages, and lymphocytes may play a major role in the damage [2].

IRI may have a negative impact on kidney transplant outcome, which is one of the main causes of delayed graft function (DGF), especially in deceased-donor kidney transplantation [3]. In addition, kidney inflammation caused by IRI may lead to increased rate of acute graft rejection [4].

Animal studies showed that an increase in plasma osmolality by infusing hypertonic saline (HS) can reduce the incidence of IRI [5-8]. In addition to having beneficial effects at the microcirculation level, infusion of HS has immune-modulator effects [9]. Infusion of HS, however, has some drawbacks such as hyperchloremic metabolic acidosis, hypernatremia, and fluid overload [10, 11]. Administration of chloride-enriched crystalloid solutions like normal saline (NS) and HS may aggravate the underlying acidosis in patients with end-stage renal disease [12].

Acidosis, apart from having adverse effects on hemodynamics, increases cytokine production and activates neutrophils [13]. Acidosis, itself, also has negative effects on renal function. For example, increased production of ammonia to adjust for the acid load in the body may cause interstitial kidney inflammation and damage [14]. Study on living donor kidney transplantation showed that intra-operative tight control of acidosis with administration of sodium bicarbonate improves early graft function [15]. However, experimental studies demonstrated that mild acidosis during early reperfusion reduces the incidence of IRI by preventing the activation of calpain enzymes and oxidative stress [16].

We therefore hypothesized that timely administration of HS, which is a hyper-osmotic agent, as well as rational use of sodium bicarbonate, which is both hyper-osmotic and anti-acidosis agent, may enhance early graft function. We designed a randomized controlled trial to evaluate the effect of bolus administration of HS, administered within renal reperfusion, and tight correction of metabolic acidosis with sodium bicarbonate, administered before and during kidney reperfusion.

## PATIENTS AND METHODS

**Patients**

All consecutive adult deceased-donor renal transplant recipients operated at transplant operating room of Sina Hospital between September 2013 and January 2015, were approached to participate in this randomized controlled trial. In the above-mentioned operating room, 10–12 renal transplantations are done monthly and more than 1000 renal transplantations, including living and deceased-donor transplants, have been performed since 10 years ago. 

The study was approved by the Ethics Committee of Tehran University of Medical Sciences. The study was registered in Iranian Registry of Clinical Trials (IRCT2013122815841N19). Inclusion criteria included first transplantation, deceased-donor kidney transplant, recipient age between 18 and 65 years, left ventricular ejection fraction of >30%, normal serum Na and K levels, panel reactive antibody of <20%, and donor serum creatinine of <2 mg/dL. Exclusion criteria included severe pre-operative or intra-operative acidosis (blood pH≤7.1 and/or serum bicarbonate level ≤10 mEq/L and/or a base excess (BE) ≤15 mEq/L), severe electrolyte abnormality ([K]<2.9 or >5.9 mEq/dL, or [Na]<125 or >145 mEq/dL), blood product transfusion, colloidal fluid administration, renal vein thrombosis, and graft anastomoses failure.

**Intervention**

All eligible patients were randomly assigned into three groups: 1) hypertonic saline, 2) sodium bicarbonate, and 3) control group. Permuted block randomization was used. Sample size calculation was based upon primary endpoints of early graft function and day three serum creatinine assuming a type one error of 0.05 and study power of 0.8 for detecting a 50% difference between studied groups, with a target of 22 patients in each group, given the standard deviations derived in our previous study [15].

Patients in the bicarbonate group were scheduled to receive 60 mL/kg of NS titrated during operation and also sodium bicarbonate 7.5% according to BE measurements. The acid-base status was measured just after induction of anesthesia and then, every 30 min up to the end of the operation. If the BE was ≤5 mEq/L in every session, the sodium bicarbonate dose was calculated as follows:

[HCO_3_] (mEq) required = 0.3 × weight (kg) × BE (mEq/L)

Half of the dose was administered through the central venous catheter during 10 min. The patients were ventilated on continuous mandatory ventilation mode according to the initial setting of RR of 10, TV of 10 mL/kg, and I/E ratio of 1/2. The ventilator setting (RR or TV) was adjusted based on patient’s PaCO_2_ level to avoid bicarbonate-induced hypercarbia and keep the PaCO_2_ between 35 and 40 mm Hg in all patients.

In the control group, about 60 mL/kg NS was titrated continuously during the operation via a central venous line to keep the central venous pressure between 10 and 15 cm H_2_O. In this group, in case of severe metabolic acidosis (BE ≤15 mEq/L or pH≤7.1), the patient was excluded from the study and treated with sodium bicarbonate infusion calculated with the same formula and catecholamine infusion, if clinically indicated. 

While 50 mL/kg of NS administered steadily to the patients in the HS group, they received 4 mL/kg HS 5% starting 10 min before graft reperfusion and lasting for about 15 min. We subtracted the amount of excess chloride given through HS infusion from that of the whole volume of NS, to avoid exacerbation of hyperchloremia in this group of patients. 

Standard monitoring according to the recommendations of American Society of Anesthesiologists was used. Radial arterial cannula was inserted before the induction of anesthesia in order to monitor blood pressure as well as obtaining blood samples for arterial blood gas analysis. General anesthesia was induced with a combination of IV midazolam (0.05 mg/kg), fentanyl (2 μg/kg), and sodium thiopental (4 mg/kg). Anesthesia was maintained using isoflurane in an air/oxygen mixture and the bolus injection of fentanyl (2 μg/kg) every hour. IV injection of atracurium (0.2 mg/kg every 20 min) provided muscle relaxation.

The immunosuppressive regimen consisted of induction with daily anti-thymocyte globulin (ATG) at a dose of 1 mg/kg for 3–7 days, followed by triple-drug immunosuppressive therapy. All patients received methylprednisolone 500 mg intra-operatively and then converted post-operatively to prednisone and tapered to 5 mg/day through several months. Mycophenolate mofetil was administered at a dose of 1000 mg twice daily, starting the day of transplantation. Cyclosporine (6 mg/kg/day twice daily) was administered after the serum creatinine level decreased to an acceptable level. All of the transplantation surgeries were performed by the same team consisted of two urologists and a vascular surgeon. 

**Study Endpoints**

The primary outcome was to compare serum creatinine levels during the first three days after operation between study groups. As the secondary outcome, we categorized graft function to immediate graft function (IGF), slow graft function (SGF), and delayed graft function (DGF). DGF was defined by need for dialysis within the first week after transplantation. SGF was defined by the serum creatinine level >2.5 mg/dL on the fifth day of kidney transplantation. Those who showed a serum creatinine <2.5 mg/dL on the fifth day were categorized as IGF [17]. Other secondary end points were urine volume in the first week, and the incidence of acute rejection during the first month after transplantation. Acute rejection was diagnosed by a nephrology team through clinical and para-clinical investigations.

Serum creatinine was measured daily until discharge of the patient. Urine volume was recorded at 2, 6, and 24 hours after transplantation, then daily within the first week after transplantation. Recipient characteristics including age, sex, weight, body mass index, base-line acid-base status, base-line hemoglobin level, cause of end-stage renal disease, length of hemodialysis, time of the last hemodialysis session before transplantation, and donor characteristics including donor age, sex, serum creatinine, cause of brain death, and cold ischemia time, were recorded.

**Statistical Analysis**

Patient demographic information and baseline characteristics were summarized using descriptive statistics. Recipient, donor and transplant parameters were compared using *Student’s t* test for independent samples or χ^2^ and Fisher’s exact tests, when appropriate. If the continuous variables did not follow a normal distribution, Mann-Whitney U test was used. Different variants of multiple measurements were separately analyzed using repeated-measure analysis. Variables with a p<0.02 on univariate analysis were included in a binary logistic regression analysis to determine independent predictors of DGF. A p value <0.05 was considered statistically significant. Analyses were conducted with SPSS^®^ for Windows^®^ ver 17 (Chicago, IL, USA).

## RESULTS

**Figure 1:**
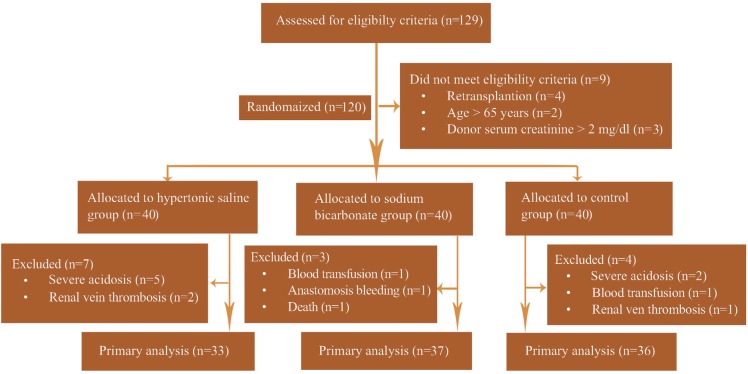
CONSORT flow chart for the study

From September 1, 2013 to January 31, 2015, a total of 129 potential deceased donor kidney transplant recipients was identified and screened for eligibility criteria. Nine patients were excluded with previous transplantation (n=4), donor serum creatinine >2 mg/dL (n=3), and age >65 years (n=2). The remaining 120 patients were randomized into three groups of HS, sodium bicarbonate, and control. The data of 37 patients in the sodium bicarbonate group, 33 in HS group, and 36 in the control group were included in the statistical analyses. Figure 1 presents the study flow chart. The baseline characteristics, intra-operative variables, and post-operative variables of recipient, donor, and transplant are summarized in Table 1. 

**Table 1:** Recipient, donor, transplant characteristics, and intra-operative variables of the studied groups. Data are presented as mean (SD), n (%), or median [range

Recipient Characteristics	Control(n=36)	Hypertonic Saline (n=33)	Bicarbonate (n=37)	p value
Age (yrs)	46 (14)	41 (10)	42 (15)	0.37
Sex (%male)	60	72	48	0.1
Weight (kg)	70.8 (19.5)	63 (10)	63 (12)	0.2
Body mass index	24.7 (4)	23.9 (4)	25.2 (4.5)	0.14
Time on pre-transplant dialysis (month)	15 [63]	24 [57]	9 [48]	0.3
Last dialysis before transplant (day)	1 [2]	1 [3]	1 [3]	0.7
Pre-operation Hb (g/dL)	9.5 (1.5)	10.2 (0.9)	9.9 (1.2)	0.46
Cause of end-stage renal disease (%)				
Diabetes	15	20	13	0.6
Hypertension	33	20	39	
Obstructive uropathy	14	8	17	
Infection	9.5	12.5	0	
Polycystic kidney	9.5	4	4	
Others	19	34.5	27	
Donor characteristics				
Age (yrs)	34(12)	30 (12)	34 (14)	0.1
Sex (%male)	76	64	62	0.4
Serum creatinine (mg/dL)	1.2 (0.35)	1.26 (0.3)	1.4 (0.3)	0.57
Cause of brain death (%)				
CVA	30	8	18	0.12
Head trauma	50	66	64	
Post-CPR	10	18	9	
Drug intoxication	10	4	4.5	
Brain tumor	0	4	4.5	
Transplant characteristics				
Cold ischemic time (hr)	4.2 (1.1)	4.26 (1.1)	4.09 (1.4)	0.72
MAP at the time of graft reperfusion (mm Hg)	103 (11)	110 (10)	110 (13)	0.3
Base-line acid-base status (after induction)				
Arterial pH	7.34 (0.06)	7.33 (0.07)	7.33 (0.07)	0.15
Base excess (mEq/L)	7.5 (3.7)	6.8 (3.3)	7.9 (3.8)	0.08
Serum bicarbonate (mEq/L)	17 (4)	18 (5)	16 (7)	0.7
PaCO_2_ (mm Hg)	37 (5)	35(7)	35(6)	0.5
Acid-base status 15 min after graft reperfusion				
Arterial pH	7.25 (0.05)	7.28 (0.01)	7.33 (0.09)	0.23
Base excess (mEq/L)	10.7 (3.2)	7.8 (3.5)	5.5 (4.0)	0.037
Serum bicarbonate (mEq/L)	15 (5)	16.9 (3)	19.6 (4)	0.7
PaCO_2_ (mm Hg)	36 (5)	38 (3)	37(7)	0.39
Saline 0.9% volume (mL/kg)	57 (23)	51 (19)	59 (15)	0.65
Chloride received during kidney transplantation (mEq/kg)	8.9 (3.7)	9.3 (4)	9.1 (2.3)	0.73
Incidence of acute rejection during first month (%)	35	24	21	0.1

CVA: Cerebrovascular accident; CPR: Cardiopulmonary resuscitation; MAP: Mean arterial pressure; ATG: Anti-thymocyte globulin; Hb: hemoglobin

**Figure 2:**
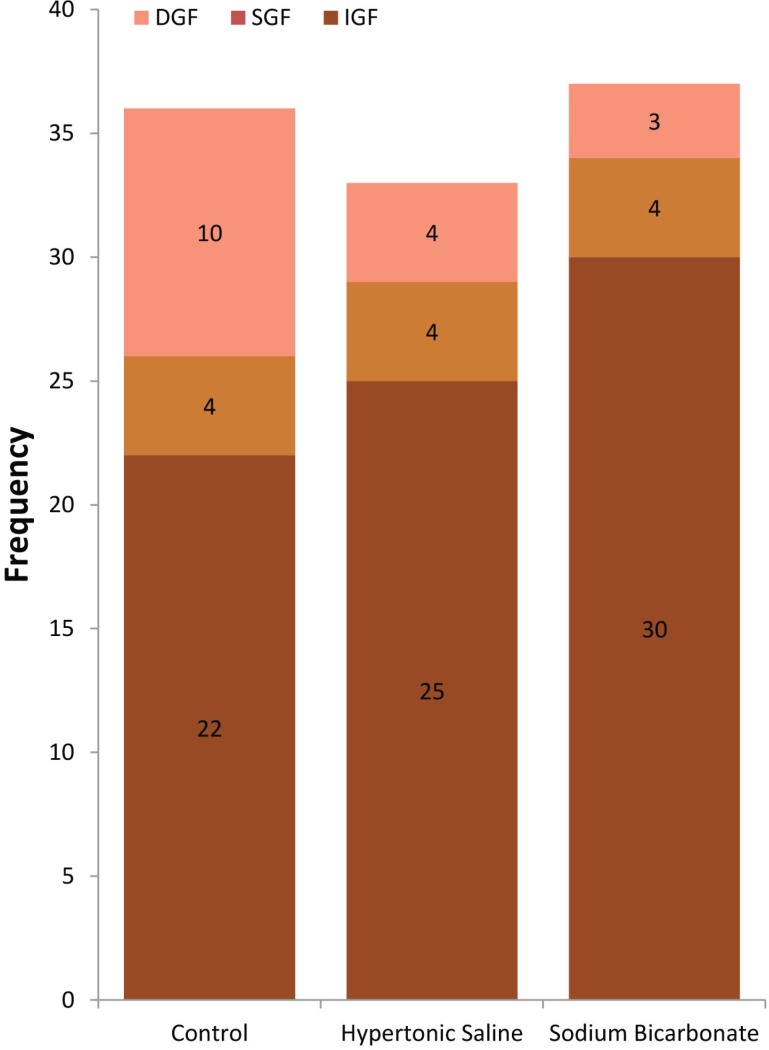
The incidence of delayed, slow, and immediate graft function between sodium bicarbonate, HS, and control groups. The rate of DGF was lower in the sodium bicarbonate and HS groups but the difference is not significant (p=0.07). DGF was defined by need for dialysis within the first week after transplantation. SGF was defined by the serum creatinine level >2.5 mg/dL on the fifth day of kidney transplantation. The remaining graft function was considered IGF. Numbers in parenthesis indicate the number of patents.

HS: hypertonic saline; DGF: delayed graft function; SDF: slow graft function; IGF: immediate graft function

The overall incidence of DGF was 17%. The incidence of DGF did not significantly differ among the three groups, though. DGF in the control group was 28% (p=0.07; Fig. 2).

**Figure 3:**
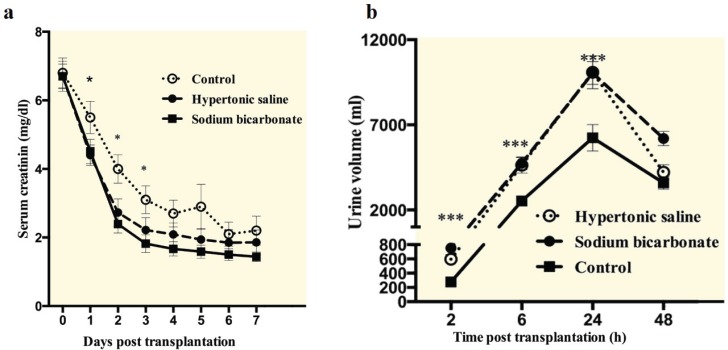
Comparison of post-transplantation serum creatinine levels and urine volumes among three studied groups. a) patients treated with sodium bicarbonate or HS, the serum creatinine levels reached <2.5 mg/dL on the second and third day of kidney transplantation, respectively. Patients in the control group had serum creatinine level <2.5 mg/dL at day 6. b) Patients who received sodium bicarbonate or HS had significantly higher urine volume compared with control group at 2, 6, and 24 hours after transplantation.

Early post-transplantation graft function was significantly improved in patients who received sodium bicarbonate or HS solution, when mean serum creatinine levels were compared at post-operative day 1 to day 3 (p<0.05). Subsequent serum creatinine values continued to be lower for the sodium bicarbonate and HS group patients, but the difference was no longer significance at days 4–7 (Fig. 3-a).

HS: hypertonic saline. *p<0.05, **p<0.01, ***p<0.001

Serum creatinine levels were not significantly different among studied groups at the hospital discharge. In patients who received sodium bicarbonate or HS, urine volume was significantly higher than that in the control group at 2, 6, and 24 hours after graft reperfusion (p<0.001). However, urine volume did not differ among the groups at the second day post-transplantation (Fig. 3-b).

Multivariate analysis of data revealed that recipient sex, age, and weight, and donor age, cause of brain death, ATG induction, and base-line arterial blood gas parameters, were not independent predictors of DGF. The rate of acute graft rejection was less in bicarbonate group in comparison to other groups but the difference was not significant (p=0.07).

## DISCUSSION

In the current study, we found that tight correction of acidosis with infusion of sodium bicarbonate or administration of HS just during graft reperfusion significantly improves early graft function up to the day three. Although the DGF rate was not significantly different between groups, considering the borderline p value obtained, this possibility should be kept in mind that if the study had been conducted in a larger sample size, we might have got a significant difference. The results of the current study supports the study conducted on living-donor transplant patients by Etezadi, *et al* [15]. 

The overall incidence of DGF was 17%, while according to the figures released by the US Renal Data System in 2009, the rate of DGF in patients undergoing deceased-donor transplantation was from 20% to 40%. Cold ischemia time, which is one of the most important risk factors for DGF, is proved to increase the risk of DGF by 10% for every six hours [18]. In our center, the time interval between kidney harvest and transplant surgery was less than eight hours. Furthermore, in our study, the lower ages of kidney donors as well as the cause of their brain death, which was mostly accidents, were also two other contributors to reduce DGF rates. It was found that the rate of DGF increases with the donor’s age; DGF rates for the ages of 20 and 65 years are 15% and 40%, respectively. According to United Network of Organ Sharing, DGF rate of the donors deceased by accidents is 18%; this rate is 30% for those who are involved in cerebrovascular events [19]. Body mass index (BMI) is regarded as a risk factor for DGF but our results did not support such a finding. 

Evidence suggests that an increase of 10 to 20 osmole in plasma osmolality, caused by HS administration, may lead to the modulation of the immune system [10]. HS has the ability to reduce accumulation of immune cells and tissue damage compared to other crystalloid solutions [20]. In human studies, it has already been found that administration of 250 mL of HS is capable of enhancing plasma sodium and osmolality from 147 to 154 and 10 to 20 mOsm, respectively. However, the increase in osmolality is temporary and begins to decline after 30 to 60 min. It is in contrast to a study that revealed plasma osmolality remains high until six hours after administration of HS [21]. It is assumed that such a temporary increase in plasma osmolality is able to affect some functions of the immune system such as production of reactive oxygen species, release of metalloproteinases, and activation of endothelial cells, while the effect persists for up to 24 hours after HS administration [22]. 

In a study conducted on a liver IRI model, HS administration just immediately before hepatic reperfusion could reduce tissue injury, while out of that time frame, it did not lead to the same beneficial effect [23]. Therefore, in our study, a HS bolus was administered immediately before the graft reperfusion in such a way that the middle of the administration coincided with the removal of renal artery clamp.

The disadvantage of chloride-rich crystalloid solutions is the induction of hyperchloremic acidosis caused by high chloride content and subsequent reduction of strong ion difference. Hyperchloremia may lead to afferent arteriole constriction, GFR reduction, and suppression of renin-angiotensin-aldosterone system which in turn, may lead to decreased urine production and sodium excretion [24]. Acidosis, itself, intensifies ammonia genesis process by the kidney. Enhanced production of ammonia leads to inflammation and kidney injury through activating alternative complement pathways [14]. Some studies have investigated the effects of different types of solutions, including NS, lactated ringer, and plasmalyte, on renal function [25-28]. However, no significant differences have been observed on renal function. Patients who received lactated ringer’s solution had greater 4-hour urine volumes and 24-hour creatinine clearance than those who received NS [27]. In addition, metabolic acidosis due to hyper-chloremia in patients who were administered NS was more profound than in those who received lactated ringer’s solution [26-28].

In the current literature, the effect of acidosis on the IRI is very controversial. Correction of acidosis may reduce IRI by modulating the immune system. On the one hand, growing evidence indicates that normal blood pH during tissue reperfusion causes exacerbation of oxidative stress, creation of mitochondrial permeability transition pore, increased apoptosis, and tissue damage [29]. Laboratory studies have demonstrated that the creation of transient acidosis (for three minutes) at the early stage of tissue reperfusion can reduce IRI. On the other hand, we know that acidosis may augment the release of NO and subsequently increases reactive oxygen species and apoptosis through PI3k-Akt-eNOS pathway [30]. Alternatively, post-ischemic conditioning has been proposed as a new strategy exerting short periods of acidosis for alleviating myocardial tissue and nervous damages caused by IRI [16]. It should be considered that the kidneys are the responsible organ for adjusting acid-base status in the body while the nervous and myocardial tissues lack such a duty. So, correction of acidosis in the blood rushed into a grafted kidney really means unloading an ischemic organ that is unable to function appropriately. It is why we got such a promising result with acidosis tight control strategy. An important concern is that we encountered five and two cases of severe metabolic acidosis in the HS and control groups, respectively. Those untoward events eventually led to the exclusion of the patients, while no patient in the bicarbonate group faced such an event. It should be noted that the only case of mortality in the bicarbonate group was due to a cardiovascular event on the fourth postoperative day. It seems that the patients in the bicarbonate group showed fewer serious complications during the study course than the other two groups.

One important finding, which needs more discussion, is that arterial blood pH and BE measured 15 min after renal reperfusion, were lower in NS-treated patients in comparison to other patients. This beneficial effect in bicarbonate group is understandable (osmotic diuresis and acidosis control due to bicarbonate therapy), but why a better acid-base status did occur in the HS group in comparison to the control group? We think it might be due to subsiding tubular cell tumescence and occurrence of earlier urinary flow and greater urine volume in these patients, which consequently led to a relative contraction alkalosis. 

In conclusion, considering the significantly improved graft function until day three together with safer therapeutic course observed in the bicarbonate group, we suggest that although both tight control of acidosis and proper administration of HS were beneficial to early renal graft function, it seems that the bicarbonate therapy is the safest strategy. Larger and probably multicenter studies are needed to reveal delayed effects of these interventions.
